# Network Construction for Bearing Fault Diagnosis Based on Double Attention Mechanism

**DOI:** 10.1155/2022/3987480

**Published:** 2022-10-29

**Authors:** QingE Wu, Tao Zong, Wenfang Cheng, Yong Li, Penglei Li

**Affiliations:** ^1^School of Electrical and Information Engineering, Zhengzhou University of Light Industry, Zhengzhou 450002, China; ^2^Polar Research Institute of China, Shanghai 200136, China; ^3^College of Mathematics and Information Science, Zhengzhou University of Light Industry, Zhengzhou 450002, China

## Abstract

Aiming at the difficulty of feature extraction in the case of multicomponent and strong noise in the traditional rolling bearing fault diagnosis method, this paper proposes a bearing fault diagnosis network with double attention mechanism. The original signal with noise is decomposed into a series of intrinsic mode functions (IMFs) by the Empirical Mode Decomposition method. The Pearson correlation coefficient is discussed to filter the IMFs components for signal reconstruction. The spatial features of the reconstructed signal are extracted by attention convolutional networks. Then, time series features are extracted based on the long short-term memory method. Furthermore, the importance of temporal features is measured through a temporal attention mechanism. The Softmax layer of the constructed network is used as the classifier for fault diagnosis. Comparing this method with the existing methods of experiments, the proposed method has not only better diagnosis accuracy but also stronger antiinterference ability and generalization ability, which can accurately diagnose and classify the bearing fault types. The fault diagnosis accuracy rate for each load is above 99%.

## 1. Introduction

As a key part of rotating machinery [1], rolling bearings are prone to failure and affect the normal operation of equipment because they work in a heavy-load, high-rotation environment for a long time [2, 3]. In order to ensure the normal operation of rotating machinery, early fault diagnosis of bearings is necessary. However, the early damage of bearings is usually small and difficult to detect, so the research on bearing fault diagnosis steps and fault feature extraction methods has become a hotspot both domestically and internationally [4, 5].

With the introduction of artificial intelligence technology, the reliability and automation of bearing fault diagnosis have been greatly improved [6]. For example, Zhang et al. [7] used an improved particle swarm optimization (PSO) algorithm to optimize variational modal decomposition (VMD) and support vector machine (SVM) for vibration signal decomposition and fault diagnosis. Experiments show that this method has high prediction accuracy, but SVM takes a longer time to calculate in the face of large sample classification. For this reason, fault diagnosis has also shifted from traditional methods to more complex methods [8]. Deep learning methods can directly process raw data, and more and more researchers use deep learning methods for classification [9]. Sun et al. [10] used wavelet transform (WT) to obtain multiscale feature signals and then used a convolutional neural network (CNN) to automatically identify fault features from multiscale signal features. Eren et al. [11] directly introduced 1D-CNN for forecasting time series, avoiding the feature extraction and feature selection process. Additionally, in order to filter out the fault feature with the largest weight from the complex signal, references [12] added an attention module to the 1D-CNN. In this paper, the features of each layer are calibrated through the multiattention convolutional neural network, thereby enhancing fault correlation and ignoring irrelevant features. In order to exploit the advantages of CNN in two-dimensional image processing, references [13] adopted a new Signal-to-Image Mapping (STIM) to convert one-dimensional vibration signals into two-bit grayscale images. Fault classification is performed by extracting features from grayscale images using CNN. The abovementioned CNNs have achieved certain results in fault classification, but they all ignore the timing of bearing fault signals. Therefore, some scholars [14] paid attention to the time series characteristics of fault signals while others paid attention to the vibration characteristics of bearing fault data. These scholars combined the advantages of CNN and long short-term memory neural network (LSTM) and designed a fault diagnosis method that uses CNN to extract high-dimensional features while LSTM considers temporal correlation. On this basis, Chen [15] proposed a CNN with different kernel sizes to extract frequency signal features and use LSTM for fault classification. The model achieved an accuracy of 98%. However, in the face of fault diagnosis of high-precision instruments, the accuracy of this model cannot meet the requirements.

In addition to the end-to-end methods of bearing fault diagnosis, there are also bearing fault diagnosis methods based on signal processing. Related scholars have proposed many different vibration signal analysis methods, namely, Spectral Kurtosis (SK) [16], Fast Kurtosis Map (FKM) [17], Minimum Entropy Deconvolution (MED) [18] and other signal analysis methods. However, in order to avoid the influence of environmental interference and obtain more satisfactory diagnostic accuracy, these signal analysis methods are usually combined with signal decomposition methods [19]. Due to the superiority of time-frequency domain analysis methods in dealing with nonstationary signals, many researchers have devoted themselves to developing fault diagnosis methods for signal characteristics in the time domain, frequency domain, and time-frequency domain [5, 20, 21]. Time-frequency analysis methods such as short time Fourier transform (STFT) [22], Wavelet Transform (WT) [23], and Empirical Mode Decomposition (EMD) [24] are continuously applied in bearing fault diagnosis. STFT is a typical time-frequency analysis method, which can convert nonstationary signals into time-frequency domain signals. Based on STFT, WT achieves both temporal resolution and frequency resolution. However, the selection of basis functions for WT is a difficult point. EMD can deal with complex nonlinear and nonstationary signals and automatically decompose complex signals into intrinsic mode functions (IMFs), where each IMF component represents the characteristics of an amplitude-modulated signal. Through the selection and reconstruction of the IMF component, the noise and other interfering signal characteristics are eliminated, and more stable and obvious signal characteristics are obtained. Therefore, some scholars have introduced EMD into intelligent algorithms for fault diagnosis. Yang [25] constructed a model based on EMD combined with a temporal convolutional network to predict the remaining useful life. This model has a higher score than the traditional CNN algorithm. However, the accumulation of convolution modules will lead to a longer model training time, which cannot meet the requirements of rapid response for bearing fault diagnosis. Wang [26] introduced EMD into the GA-BP algorithm for fault diagnosis of rolling bearings but also faced the problem of slow learning speed. In order to solve the influence of strong background noise and other disturbances, a novel bearing fault information-guided VMD (FIVMD) method has been proposed by Ni [27] to extract the weak bearing repetitive transients. Experiments demonstrate that the proposed FIVMD is a superior method for extracting weak-bearing repetitive transients. The authors in [28] proposed a brand-new health indicator (HI), which can reflect the changes in the probability distribution of all cyclic power spectra over time. Any form of variation in modulation characteristics can be revealed through the HI, even the weak information buried by the internal or external noise. The abovementioned algorithms have a certain effect on bearing fault diagnosis, but bearing fault is a complex problem, and the effect of the above algorithms is not clear in the case of load variation.

In view of the abovementioned problems, in order to obtain a bearing fault diagnosis model with fast diagnosis speed, high diagnosis accuracy, and strong generalization ability, this paper proposes a bearing fault diagnosis network with double attention mechanism. First, this paper uses EMD to decompose the bearing fault data. As a method for analyzing nonlinear and nonstationary data, EMD can decompose any complex dataset into a limited and usually small number of IMF components. Spurious harmonics of nonlinear and nonstationary signals are eliminated by the selection and reconstruction of IMF components. Bearing fault signals are nonlinear signals containing a lot of noise. Therefore, as a data-driven adaptive nonlinear signal decomposition method, EMD is the most suitable for processing nonlinear signals of rolling bearings [29, 30]. Then, the Pearson correlation coefficient is used to filter out the signal with a strong correlation with the original signal for reconstruction. Spatial features of the reconstructed signal are extracted by attention convolutional networks. LSTM is used to extract time series features, and the time series feature attention mechanism is added to the hidden layer of LSTM. The improved LSTM can distinguish the time information of different historical moments and assign different time attention weights to the information of different historical moments according to their influence on this moment. The Softmax layer of the constructed network is used to classify the bearing state. In this model, the interference signal is removed by EMD, and the spatial and temporal features are extracted through the attention convolutional neural network and LSTM, respectively. The importance of temporal features is distinguished by the temporal attention mechanism, thereby realizing the multilevel feature extraction of the model. From the experimental results, the diagnostic accuracy of the model is improved to a certain extent compared with the previous models, and it has good generalization ability and robustness.

## 2. Design of Bearing Fault Diagnosis Network Based on Dual Attention Mechanism

### 2.1. Model Establishment

**Figure 1:**
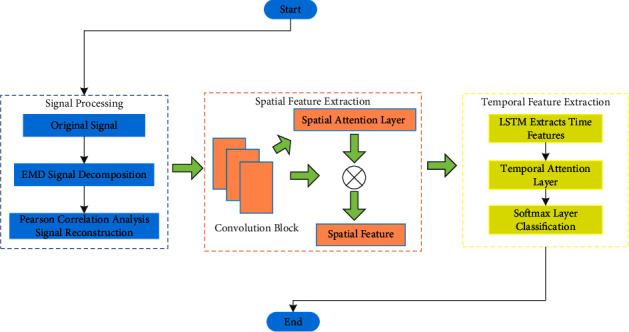
Flowchart of bearing fault diagnosis for double attention mechanism.

The bearing fault diagnosis network model of the double attention mechanism not only considers the spatial characteristics of bearing fault signals but also considers the temporal correlation of fault signals. The Empirical Mode Decomposition (EMD) method can eliminate a large amount of noise mixed in the original bearing signal to filter out the interference signal in the original signal and enhance the antiinterference ability of the model. Although the traditional convolutional neural network (CNN) model can extract spatial features, it is difficult to distinguish the contribution of the extracted features to the classification task, and the pooling layer will lead to the loss of some feature information while reducing the dimension. Therefore, this model uses the spatial feature attention mechanism to replace the traditional pooling layer. The constructed attention convolution network can not only retain the integrity of the extracted features but also give different degrees of attention to different features by distributing weights. The attention convolution network has a good effect on spatial feature extraction, but it is not sensitive to time series features. It is difficult to capture the connection before and after a time series. LSTM can learn the time correlation of time series information through different gating units, which makes up for the shortcomings of attention convolution networks in time series feature extraction. In this paper, the attention convolutional network is combined with LSTM, so that the model can extract features in both spatial and temporal dimensions at the same time. Similarly, a temporal attention mechanism is added after the LSTM hidden layer to highlight the historical information that has a greater impact on the current time period and suppress the less influential or useless historical information, so that the model's antiinterference ability is further strengthened. The flowchart of the established bearing fault diagnosis model is shown in Figure 1.

### 2.2. Decomposition Method of Fault Signal Based on EMD

EMD was proposed by Huang et al. [29] first, which is a time-frequency processing method that is very suitable for processing nonstationary signals [31]. It can decompose the original signal into a series of Intrinsic Mode Functions (IMFs), and each IMF signal needs to meet the following two conditions: in the entire time range, the difference between the number of local extreme points and the number of zero-crossing points of the function is at most one; at any time, the mean of the two upper and lower envelopes of the local maximum point and the local minimum point of the function must be 0. The specific decomposition process is as follows:

(1) We determine all the maximum and minimum points of the original signal *x*(*t*), use the cubic spline interpolation method to fit the obtained maximum and minimum points to form the upper envelope *y*_up_(*t*) and the lower envelope line *y*_low_(*t*), and then calculate the average envelope line avg_1_(*t*) of the signal.
  (1) $$\begin{array}{c} Avg_{1}\left(t\right)=\frac{y_{up}\left(t\right)+y_{low}\left(t\right)}{2}\text{.} \end{array}$$
(2) The average envelope signal avg_1_(*t*) is subtracted from the original signal *x*(*t*); that is, the first new signal *h*_1_(*t*) with the low frequency removed is obtained.
  (2) $$\begin{array}{c} h_{1}\left(t\right)=x\left(t\right)-avg_{1}\left(t\right)\text{.} \end{array}$$
(3) e determine whether *h*_1_(*t*) satisfies the IMF condition. If so, then *h*_1_(*t*) is the first-order IMF component of *x*(*t*). Otherwise, *h*_1_(*t*) is regarded as a new signal to be decomposed, and steps (1) and (2) are executed cyclically until the first component that meets the IMF characteristic conditions is obtained, which is denoted as IMF_1_.
(4) We separate the first IMF component containing high-frequency signal components from the original signal *x*(*t*) to obtain *R*_1_ as follows:

(3) $$\begin{array}{c} R_{1}\left(t\right)=x\left(t\right)-IMF_{1}\left(t\right)\text{.} \end{array}$$

Take *R*_1_(*t*) as a new original signal, and repeat the above steps. The second IMF component of the original signal *x*(*t*) can be obtained, which contains lower frequency components than IMF_1_. Repeat the above operations to obtain IMF_1_, IMF_2,_ ⋯ , IMF_*n*_, respectively, until when the residual component *R*_*n*_(*t*) is a monotonic function or very small, and the decomposition process ends.

At this point, the result of the original signal *x*(*t*) after EMD decomposition can be obtained.

(4) $$\begin{array}{c} x\left(t\right)=\overset{n}{\underset{i=1}{\sum }}IMF_{i}\left(t\right)+R_{n}\left(t\right)\text{.} \end{array}$$

In the formula, the IMF component contains the frequency components of the original signal *x*(*t*) in different frequency bands, which can accurately reflect the local feature information of the original signal at different scales. The residual component *R*_*n*_(*t*) represents the central trend of the original signal *x*(*t*).

### 2.3. Spatial Feature Extraction Method Based on Convolutional Neural Network

As a multilayer neural network, a convolutional neural network usually includes convolutional layer, pooling layer, and fully connected layer and is widely used in image processing, natural language processing, speech recognition, and other fields. The convolution layer extracts the hidden feature information in the data through the convolution operation and enhances the network expression ability through the nonlinear activation function. The operation process of the convolution is as follows:

(5) $$\begin{array}{c} x_{j}^{l}=f\left(\overset{M}{\underset{i=1}{\sum }}x_{i}^{l-1}\times k_{ij}^{l}+b_{j}^{l}\right)\text{.} \end{array}$$

In the formula, *x*_*j*_^*l*^ is the *jth* feature map of the *lth* layer, *f*(•) is the nonlinear activation function, *M* is the number of input feature maps, *x*_*i*_^*l*−1^ is the *ith* feature map of the (*l* − 1)*th* layer, *k*_*ij*_^*l*^ is the convolution kernel, and *b*_*j*_^*l*^ is the bias of the *jth* feature map of the *lth* layer.

**Figure 2:**
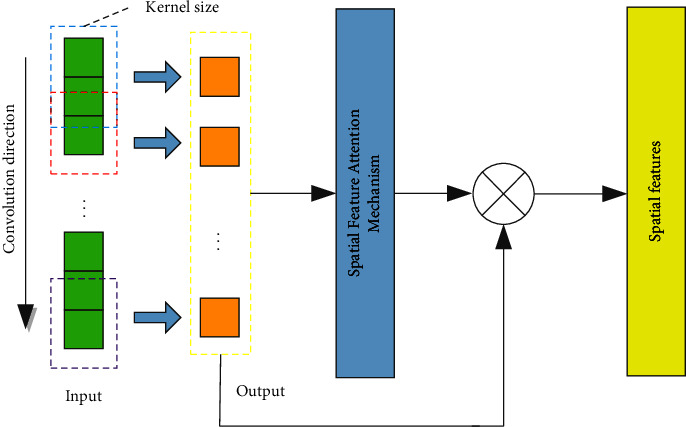
Attention convolutional network architecture.

The pooling layer usually includes max pooling and average pooling, which improves the efficiency of model operations by integrating feature information and reducing dimensionality. However, performing max pooling or average pooling in each region of this model inevitably discards some features, which leads to information loss and affects the accuracy of fault diagnosis. Therefore, this paper constructs an attention to convolution network and uses the spatial attention mechanism to replace the pooling layer. The network uses the attention mechanism to assign weights to the features to distinguish the importance of the features. Its working mechanism is to transfer the spatial features extracted by the convolution block to the spatial feature attention mechanism and use the attention mechanism to give less weight to the features that contribute less to the classification task instead of directly discarding them. A larger weight is given to the feature with a larger contribution to the classification task to strengthen the model's attention to this feature. The obtained weights are weighted with the features extracted by the convolution block to obtain the final spatial features. Compared with traditional convolutional neural networks, the attention convolutional network preserves the integrity of information while highlighting important features. The constructed attention convolutional network is shown in Figure 2.

The fully connected layer is usually placed at the end of the model to integrate the features extracted by the model to prepare for subsequent regression or classification.

### 2.4. Feature Weighting Method Based on Attention Mechanism

The attention mechanism is a resource allocation mechanism borrowed from human attention [32], which can adaptively assign attention weights to input features and assign more weights to key features. In the neural network model, the attention mechanism can be used to select the features that are most helpful to the task goal of the model among many features, so that the model can achieve better results. The attention mechanism is divided into soft attention mechanism and hard attention mechanism according to the area of attention in each step [33]. The soft attention mechanism considers all inputs and will focus on some specific inputs, while the hard attention mechanism is a random process that only pays attention to one input at a time.

This paper adopts the soft attention mechanism, and the specific process is divided into the following three steps: First, the similarity calculation is performed on all input vectors *X*=[*x*_1_, *x*_2_, ⋯, *x*_*n*_] to obtain the attention value *A*_*n*_. Then, the attention value *A*_*n*_ is normalized by the Softmax function to obtain the attention weight. Finally, all input vectors and the corresponding weight coefficients are weighted to obtain the vector of the original input vector after the attention weighting.

### 2.5. Time Series Feature Extraction Method Based on Long Short-Term Memory Network

**Figure 3:**
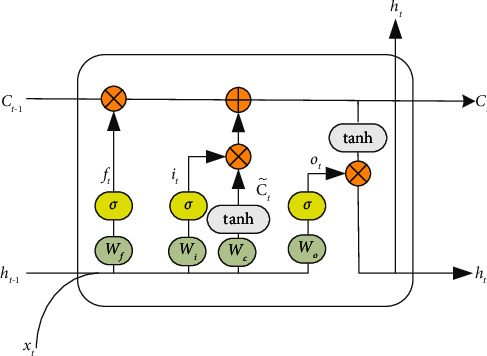
LSTM network structure.

LSTM [34] is an improvement to solve the problem of gradient disappearance and gradient explosion in the training process of long sequence samples of Recurrent Neural Network (RNN). Different from RNN, LSTM adds a gating unit to each neuron to measure the importance of historical information to the current moment. The gating unit LSTM can retain and transmit long-term time series information, which solves the problem of gradient disappearance and gradient explosion in the traditional RNN training process. The structure of the LSTM model is shown in Figure 3.

As shown in the figure, the LSTM network is mainly composed of a forget gate, an input gate, and an output gate. The forget gate is responsible for selectively discarding historical information, the input gate retains the current external information and fuses it with historical information, and the output gate determines the impact of the current gating unit state on the output of the hidden layer. In addition, the gating unit is the core of the LSTM network, which is responsible for the overall planning and transmission of information, and updates the parameters according to the results of the forget gate and the input gate. The final output of the LSTM network is jointly determined by the output gate and the gating unit, which can be expressed as follows:

(6) $$\begin{array}{c} f\left(t\right)=\sigma \left(W_{f}h_{t-1}+U_{f}x_{t}+b_{f}\right)\text{,}\\ i\left(t\right)=\sigma \left(W_{i}h_{t-1}+U_{i}x_{t}+b_{i}\right)\text{,}\\ o\left(t\right)=\sigma \left(W_{o}h_{t-1}+U_{o}x_{t}+b_{o}\right),\\ \overset{˜}{C}\left(t\right)=\tanh\;\left(W_{\overset{˜}{C}}h_{t-1}+U_{\overset{˜}{C}}x_{t}+b_{\overset{˜}{C}}\right),\\ C\left(t\right)=C\left(t-1\right)⊗f\left(t\right)+i\left(t\right)⊗\overset{˜}{C}\left(t\right),\\ h\left(t\right)=o\left(t\right)⊗\tanh\;\left(C\left(t\right)\right). \end{array}$$

In the formula, *h*_*t*−1_ is the hidden layer state vector at time *t* − 1, *x*_*t*_ is the input vector at time *t*, *W*_*f*_, *U*_*f*_, and *b*_*f*_, *W*_*i*_, *U*_*i*_, and *b*_*i*_, *W*_*o*_, *U*_*o*_, and *b*_*o*_, and $W_{\overset{˜}{C}}$, $U_{\overset{˜}{C}}$, and $b_{\overset{˜}{C}}$ are the weights and biases of the forget gate, input gate, and output gate, respectively, *σ* is the sigmoid activation function, tanh is the hyperbolic tangent activation function, and ⊗ is the matrix multiplication.

### 2.6. Dual Attention Mechanism Bearing Fault Diagnosis Model Learning

**Figure 4:**
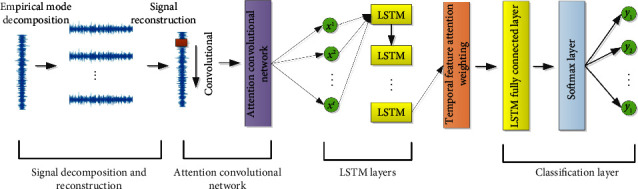
Model learning process.

The bearing fault diagnosis network of the dual attention mechanism is mainly composed of four modules, the signal decomposition and reconstruction module, the attention convolutional network module, the LSTM module, and the Softmax layer classification module. Its structure is shown in Figure 4.

As shown in the figure, EMD is used to decompose the collected bearing signal, and a series of IMFs after decomposition are analyzed by Pearson correlation coefficient. The IMF components with strong correlation with the original signal are screened out for signal reconstruction to eliminate the influence of noise. The Pearson correlation coefficient can measure the strength of the linear relationship between two variables, and it has the advantages of a clear analytical expression and a simple calculation program [35]. Therefore, in this paper, the Pearson coefficient is used to measure the correlation between the IMF components and the original signal, and the IMF components with strong correlation with the original signal are screened out. The Pearson correlation coefficient is calculated as follows:

(7) $$\begin{array}{c} Pearson=\frac{\overset{n}{\underset{i=1}{\sum }}\left(x_{i}-\bar{x}\right)\left(y_{i}-\bar{y}\right)}{\sqrt{\overset{n}{\underset{i=1}{\sum }}{\left(x_{i}-\bar{x}\right)}^{2}}\sqrt{\overset{n}{\underset{i=1}{\sum }}{\left(y_{i}-\bar{y}\right)}^{2}}}\text{.} \end{array}$$

Among them, *X*=(*x*_1_, *x*_2_, ⋯, *x*_*i*_, ⋯, *x*_*n*_) and *Y*=(*y*_1_, *y*_2_, ⋯, *y*_*i*_, ⋯, *y*_*n*_) are two vectors. $\bar{x}$ and $\bar{y}$ are the sample mean. The value range of the Pearson correlation coefficient is [−1, 1], the closer to 0, the weaker the correlation, and the closer to 1, the stronger the positive correlation, the closer it is to −1, the stronger the negative correlation.

**Figure 5:**
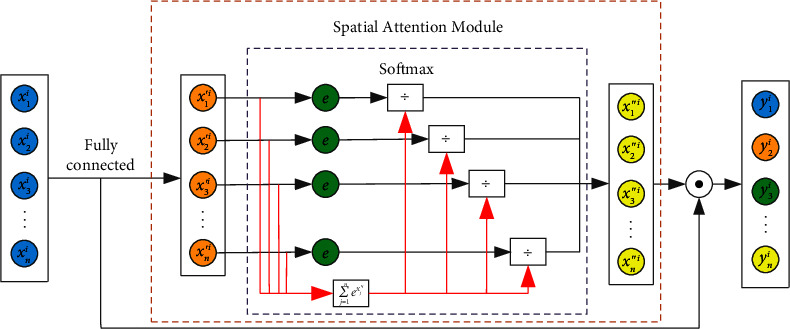
Fault space feature attention mechanism model.

For the reconstructed fault signal, attention convolutional network is used for spatial feature extraction, which is composed of two convolution modules and a spatial attention mechanism. The convolutional layer is used to extract spatial features, and the spatial feature attention mechanism implements weighting of different spatial features. Compared with the traditional convolutional neural network, this model overcomes the disadvantage of information loss in the pooling layer and adaptively assigns different weights to different features through the attention mechanism to highlight the influence of important features and reduce or ignore the influence of irrelevant features. The structure diagram of spatial feature attention mechanism is shown in Figure 5.

As shown in the figure, the feature vector *x*^*i*^=[*x*_1_^*i*^, *x*_2_^*i*^, *x*_3_^*i*^, ⋯, *x*_*n*_^*i*^] is the feature vector containing *n* features extracted by the convolution block module at the *ith* time step, and the attention weight vector *x*′^*i*^=[*x*′_1_^*i*^, *x*′_2_^*i*^, *x*′_3_^*i*^, ⋯, *x*′_*n*_^*i*^] is obtained through a single-layer neural network.

(8) $$\begin{array}{c} {x^{\prime }}^{i}=\tanh\;\left(W_{{x^{\prime }}^{i}}x^{i}+b_{{x^{\prime }}^{i}}\right)\text{.} \end{array}$$

Among them, *W*_*x*′^*i*^_ is the trainable weight matrix, *b*_*x*′^*i*^_ is the bias vector for calculating the feature attention weight, and tanh(•) is the activation function.

Then, the attention weight vector *x*′^*i*^ is normalized with the Softmax function to obtain the attention weight coefficient *x*^″^^*i*^=[*x*^″^_1_^*i*^, *x*^″^_2_^*i*^, *x*^″^_3_^*i*^, ⋯, *x*^″^_*j*_^*i*^, ⋯, *x*^″^_*n*_^*i*^]

(9) $$\begin{array}{c} {x^{\prime \prime }}_{j}^{i}=\frac{e^{{x^{\prime }}_{j}^{i}}}{\overset{n}{\underset{m=1}{\sum }}e^{{x^{\prime }}_{m}^{i}}}\text{.} \end{array}$$

Among them, *x*^″^_*j*_^*i*^ is the normalized spatial attention weight coefficient of the *jth* feature of the spatial feature attention weight vector *x*′^*i*^.

Therefore, the spatial feature vector *y*^*i*^=[*y*_1_^*i*^, *y*_2_^*i*^, *y*_3_^*i*^, ⋯, *y*_*n*_^*i*^] after weighted by the attention mechanism can be obtained.

(10) $$\begin{array}{c} y^{i}=x^{i}⊙{x^{\prime \prime }}^{i}=\left[x_{1}^{i}\cdot {x^{\prime \prime }}_{1}^{i},x_{2}^{i}\cdot {x^{\prime \prime }}_{2}^{i},x_{3}^{i}\cdot {x^{\prime \prime }}_{3}^{i},\cdots ,x_{n}^{i}\cdot {x^{\prime \prime }}_{3}^{i}\right]\text{.} \end{array}$$

In the formula, ⊙ represents the Hadamard product.

**Figure 6:**
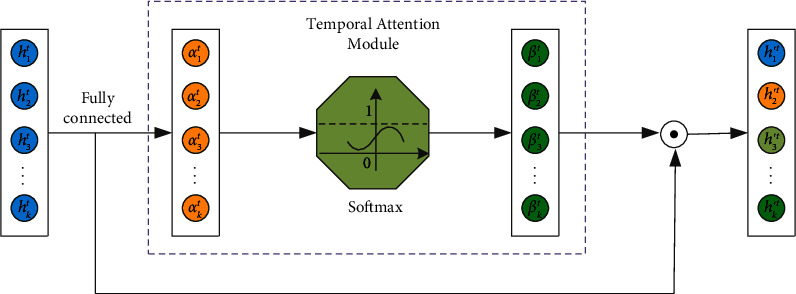
A model of attention mechanism for fault temporal features.

The vector after the spatial attention mechanism is directly sent to the LSTM layer, and the four-layer LSTM network is used to capture the temporal dependencies of the features. The dropout layer is embedded in the LSTM, so that the model ignores a certain number of neurons with a certain probability and achieves the effect of regularization to a certain extent. Similarly, the correlation between the information of each historical moment and the information of the current moment in the extracted time series features is mined through the temporal attention mechanism, and different temporal attention weights are given to them according to their contribution degrees to enhance the influence of important temporal features in the task target. The structure diagram of the temporal attention mechanism is shown in Figure 6.

As shown in the figure, the input of the temporal feature attention mechanism is the temporal feature *h*^*t*^=[*h*_1_^*t*^, *h*_2_^*t*^, *h*_3_^*t*^, ⋯, *h*_*k*_^*t*^] learned by the LSTM network iteratively to the time *t*, where *k* is the length of the input sequence time window. Then, the temporal feature attention weight vector at the current moment is *α*^*t*^=[*α*_1_^*t*^, *α*_2_^*t*^, *α*_3_^*t*^, ⋯, *α*_*k*_^*t*^].

(11) $$\begin{array}{c} \alpha^{t}=\tanh\;\left(W_{\alpha^{t}}h^{t}+b_{\alpha^{t}}\right)\text{.} \end{array}$$

Among them, *W*_*α*^*t*^_ is the trainable weight matrix, *b*_*α*^*t*^_ is the bias vector for calculating the feature attention weight, and tanh(•) is the activation function.

In the same way, use Softmax to normalize *α*^*t*^ to get the temporal feature attention weight coefficient as *β*^*t*^=[*β*_1_^*t*^, *β*_2_^*t*^, *β*_3_^*t*^, ⋯, *β*_*j*_^*t*^, ⋯, *β*_*k*_^*t*^].

(12) $$\begin{array}{c} \beta_{j}^{t}=\frac{e^{\alpha_{j}^{t}}}{\overset{k}{\underset{m=1}{\sum }}e^{\alpha_{m}^{t}}}\text{.} \end{array}$$

Among them, *β*_*j*_^*t*^ is the normalized temporal attention weight coefficient of the *jth* feature of the temporal feature attention weight vector *α*^*t*^.

The temporal feature attention weight is weighted with the temporal features of each historical moment to obtain the comprehensive time series information feature *h*′^*t*^=[*h*′_1_^*t*^, *h*′_2_^*t*^, *h*′_3_^*t*^, ⋯, *h*′_*k*_^*t*^].

(13) $$\begin{array}{c} {h^{\prime }}^{t}=h^{t}⊙\beta^{t}=\left[h_{1}^{t}\cdot \beta_{1}^{t},h_{2}^{t}\cdot \beta_{2}^{t},h_{3}^{t}\cdot \beta_{3}^{t},\cdots ,h_{k}^{t}\cdot \beta_{k}^{t}\right]. \end{array}$$

Finally, all the weighted hidden layer states *h*′^*t*^ are sent to the Softmax layer to realize the fault diagnosis of rolling bearings.

## 3. Model Validity Experiment and Result Analysis

This paper used the experimental data of the Bearing Data Center of Case Western Reserve University in the United States to test the effectiveness of the method in this paper. The experiment is implemented based on MATLAB and TensorFlow deep learning framework.

### 3.1. Data Sources and Dataset Creation

In this experiment, the vibration data of SKF6205 drive end rolling bearing of Case Western Reserve University in the United States are selected, the sampling frequency is 48 Khz, and the speed is 1771r/min. This dataset has a variety of different load types, namely, 0 hp, 1 hp, 2 hp, and 3 hp. In this experiment, the data under the 1 hp load condition are selected to train the model, and other loads are used to verify the generalization and migration ability of the model. There are three types of faults in this dataset: rolling element faults, outer ring faults, and inner ring faults, each of which is divided into three different sizes: 0.007, 0.014, and 0.021 (1 in equals 0.0254 m), so this model has nine fault states plus one normal state, which is a ten-class problem. In this paper, 1024 consecutive points were taken as a data sample, and a total of 4640 samples are obtained. The specific experimental samples and fault labels are shown in Table 1.

### 3.2. Fault Diagnosis Experiment under 1 hp Load

**Figure 7:**
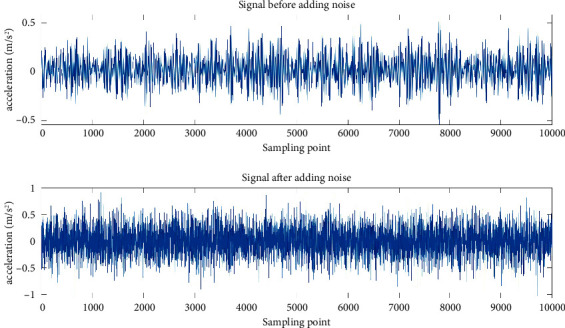
Original and noisy signals.

Due to the complexity of the bearing working environment, the bearing fault data collected under actual working conditions usually contain a lot of noise. For this reason, before the experiment, this paper added Gaussian white noise on the basis of the original signal, the signal-to-noise ratio is SNR=−5, and the rolling element fault with a fault diameter of 0.007 was used as a demonstration example. The original signal and the signal with added noise are shown in Figure 7, and only 10,000 sampling points are shown for the convenience of observation.

**Figure 8:**
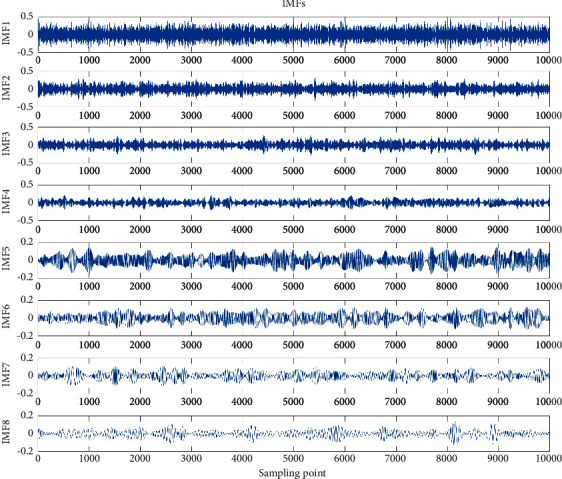
IMF components after rolling bearing fault with fault diameter of 0.007.

It can be seen from the figure that the signal after adding Gaussian white noise is more complex, which can more truly reflect the real situation of the bearing signal. EMD method is used to decompose the noise added signal into IMF components, and then the Pearson correlation coefficient method was used to screen out the IMF components whose correlation coefficient is greater than 0.1 with the original signal. For the IMF whose correlation coefficient is less than 0.1, the correlation with the original signal is extremely weak, so this paper discarded them. The IMF components were screened for the rolling element fault with a fault diameter of 0.007 shown in Figure 8 and the first 10,000 sampling points are also displayed in this paper for the convenience of comparison.

As shown in the figure, the fault information contained in IMF_8_ has been relatively sparse, so it is feasible to use the Pearson correlation coefficient screening method to screen the IMF components, which can screen out the decomposition signals containing a large amount of fault information. For the filtered IMF components, this paper reconstructed them as follows:

(14) $$\begin{array}{c} signal=\overset{n}{\underset{i=1}{\sum }}IMF_{i}\text{.} \end{array}$$

In the formula, *n* represents the number of all IMF components whose correlation coefficient with the original signal is greater than 0.1.

Before the signal is imported, this paper first sets the parameters of the model. The number of model training was initially set to 100 times. The Batch size was set to 64. The training optimizer chose Adam. The loss function was the cross entropy loss function. The convolution kernel size of the convolution layer was set to 3 × 1, and the step size was set to 1. The hyperbolic tangent activation function was selected as the nonlinear activation function in the attention convolutional networks and the LSTM network. The specific parameters of the model structure are shown in Table 2.

**Figure 9:**
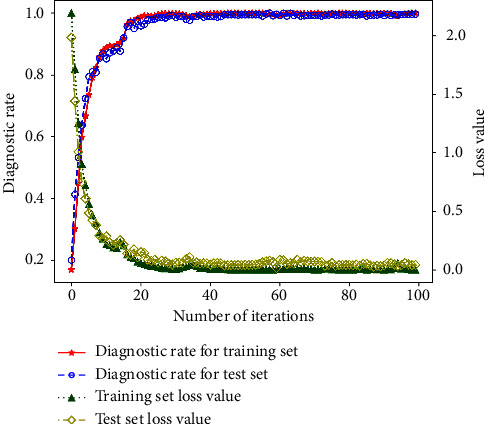
Fault diagnosis results by this proposed model.

The reconstructed signal was imported into the network, and the accuracy of the training set and test set of the model under 1 hp is shown in Figure 9. With the addition of white Gaussian noise, the classification accuracy of this model on the training set reaches 100%. On the test set, the classification accuracy can reach 99.78%, and the loss value converges to around 0.0205. In addition, the convergence speed of this model can also achieve satisfactory results. It can be seen from the figure that the prediction accuracy of this model increased almost linearly, and the loss function decreased linearly. After about 20 iterations, the model accuracy basically remains above 99%, and the loss function also remains at a low level. Therefore, it can be concluded that the model not only has satisfactory results in the diagnosis rate but also has excellent performance in stability and convergence speed.

**Figure 10:**
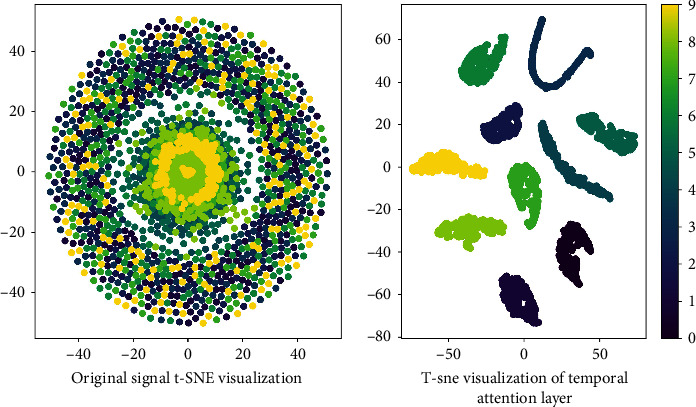
T-SNE for feature visualization.

In order to intuitively illustrate the feature extraction capability of this model, Figure 10 used t-distributed Stochastic Neighbor Embedding (t-SNE) to visualize the original signal features and the features extracted by this model. As can be seen from the t-SNE diagram of the original signal, the characteristics of the original signal are very chaotic and the clustering effect is poor. The features obtained after the temporal attention layer have been basically divided into ten categories, which have a good clustering effect. Different clustering results prove the excellent performance of this model in feature extraction.

**Figure 11:**
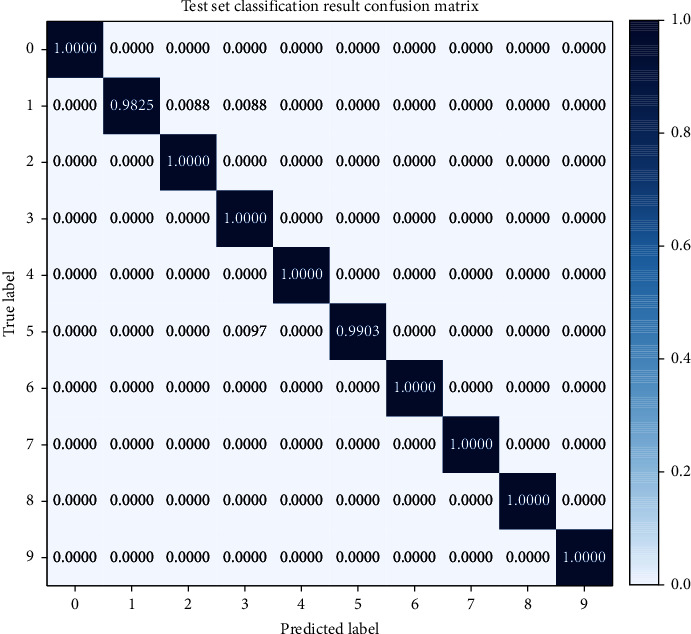
The results of the model's test classification for each state of the bearing.

In addition, in order to specifically analyze the diagnostic effect of this model for each type of fault, Figure 11 shows the confusion matrix of the classification results of this model, where the abscissa and ordinate represent the predicted label and the true label, respectively. It can be seen from the confusion matrix that this model only had a small amount of errors in the diagnosis of labels 2 and 5. The model in this paper divides 0.88% of the first type of faults into the second type of faults, 0.88% of the first type of faults into the third type of faults, and 0.97% of the fifth type of faults into the third type of faults; the diagnosis of other labels had reached 100% correct rate. So, it can be concluded that the model can effectively extract the characteristics of fault information for each type of fault signal and can effectively identify and classify these characteristics.

### 3.3. Ablation Experiments of the Proposed Model

In order to illustrate the role of each part of the model constructed in this paper, this paper designs an ablation experiment, which deletes different modules of the model to explore the role of each module in feature extraction. Models for ablation experiments include attention convolutional networks, attention-weighted LSTM, 1D-CNN-LSTM, and double attention model lacking EMD denoising. All parameters of these models are identical except for the different structures.

**Figure 12:**
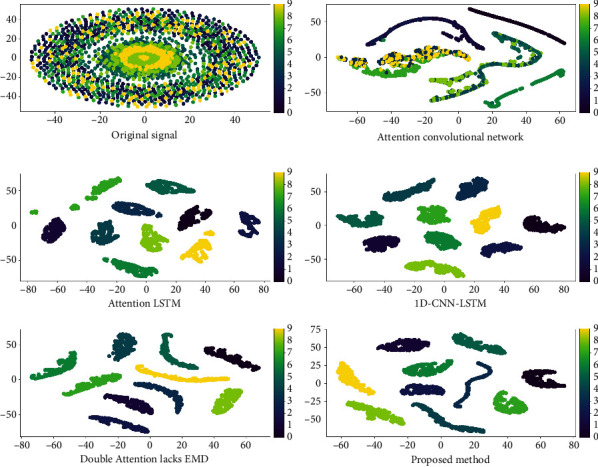
t-SNE visualization results of extracted features for each model.

Figure 12 is the result of dimension reduction visualization of feature vectors extracted from different models by t-SNE algorithm. After the fault information is extracted by attention convolution, there are still many features that have not been separated. The bearing fault signal contains a lot of time series features, and the convolutional neural network is less sensitive to the time series features, which leads to a poor feature extraction effect. The features extracted by the attention-weighted LSTM show a clustering trend; however, it does not divide the features into 10 categories because of the low sensitivity of LSTM to spatial features. 1D-CNN-LSTM, double attention model lacking EMD denoising, and the model constructed in this paper consider both spatial and temporal features. Therefore, they have similar effects in feature extraction, and they can all classify samples into ten categories.

**Figure 13:**
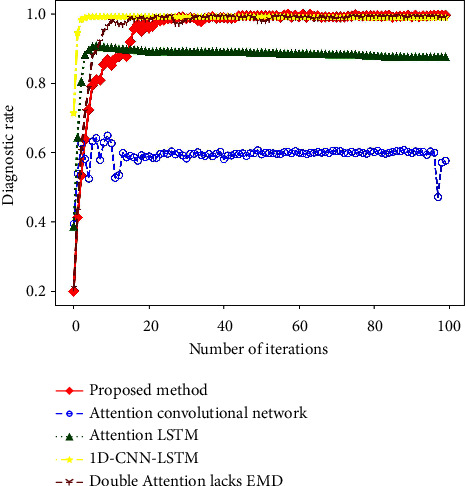
Fault diagnosis results of each model in ablation experiments.

The diagnostic process of each model is shown in Figure 13. Due to the poor feature extraction effect of the attention convolutional network, the model diagnosis rate is low, and the diagnosis accuracy rate is only 60%. Because of its high sensitivity to time series features and insensitivity to spatial features, the prediction accuracy of LSTM can only reach 87.42%. 1D-CNN-LSTM can extract time series features and spatial features at the same time, and its accuracy rate reaches 98.57%. CNN and LSTM are improved through the attention mechanism, which strengthens the model's attention to important features, so the diagnosis rate of double attention network without EMD noise reduction reaches 99.24%. The model proposed in this paper uses EMD to denoise the original signal on the basis of the double attention mechanism, which can filter out the interference in the original signal and further improve the accuracy of the model. Therefore, the diagnostic rate of the model proposed in this paper is the highest among all models, reaching 99.78%. The fault diagnosis results of each model are shown in Table 3. All diagnostic results are the average of 10 replicates. It can be seen that the diagnostic accuracy of this model is the highest, which is 39.44%, 12.36%, 1.21%, and 0.54% higher than the other four models.

## 4. The Comparison between the Proposed Model and the Existing Model Methods

### 4.1. Comparison of Experimental Results between the Proposed Model and the Existing Model

In order to highlight the diagnostic performance of the model in this paper, the SSA-SVM [36], PSO-RF [37], and AVSSA-KELM [38] models are selected as experimental comparison models, which are established based on temporal feature extraction. SSA-SVM uses the SSA optimization algorithm to find the optimal penalty coefficient and kernel function parameters of the SVM, so that the SVM fault diagnosis rate is improved. PSO-RF uses the PSO algorithm to optimize the random forest to find the relationship between the algorithm performance and the number of decision trees and find the optimal number of decision trees. AVSSA-KELM uses AVSSA to optimize the kernel parameters and regularization coefficients of KELM, so that the classification effect of KELM can be optimized.

**Figure 14:**
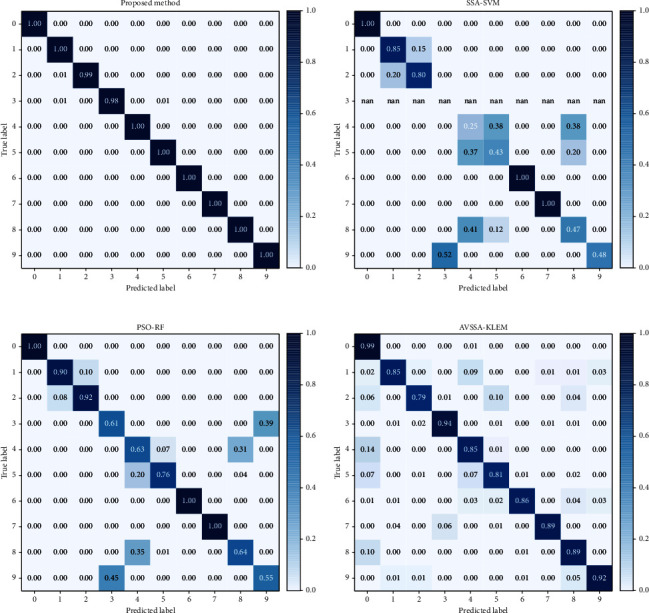
Diagnosis results between the model in this paper and the existing model.

The diagnostic results of each model are shown in Figure 14. The model constructed in this paper has a 100% diagnosis rate for almost every fault of the bearing, which is significantly improved compared with SSA-SVM, PSO-RF, and AVSSA-KELM. The total accuracy of the comparison model and the model proposed in this paper for 10 kinds of fault diagnosis is shown in Table 4. Faced with the problem of bearing fault diagnosis, the optimized machine learning algorithm's diagnosis effect is still not as good as the model in this paper. The diagnostic rate of the model in this paper reaches 99.78%, which is 29.2%, 20.15%, and 11.77% higher than that of SSA-SVM, PSO-RF, and AVSSA-KELM, respectively, which can fully meet the actual bearing fault diagnosis.

### 4.2. The Generalization Ability Test Analysis of the Proposed Model and the Existing Models

**Figure 15:**
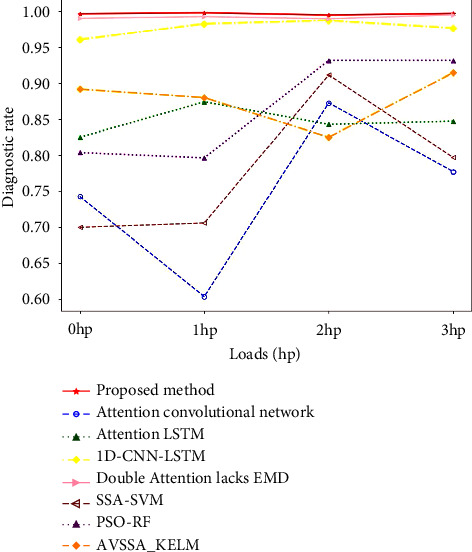
Comparison of the generalization ability of this model and existing models.

It can be seen from the above experiments that this model can achieve good accuracy for bearing fault diagnosis under 1 hp load. In order to verify the generalization ability of the model, this paper tested the bearing data under 0, 2, and 3 hp loads, respectively. The sampling of the data set is unchanged, the division of the training set and the test set is the same as the 1 hp load, and the diagnosis results in this paper are compared with the models of the ablation experiment and the machine learning comparison models. The diagnostic results of different models are shown in Figure 15. The diagnostic rate of the model proposed in this paper is close to 100% under various loads, and the diagnostic effect is significantly higher than all the comparative models. Therefore, it can be shown that this model has more advantages than traditional algorithms in bearing fault diagnosis, and the diagnosis results are more reliable. To more directly illustrate the diagnostic performance of these models, the diagnostic rate of the models under each load and the average diagnostic rate under four loads are presented in Table 5. The diagnostic rates of the model constructed in this paper are 99.64%, 99.78%, 99.46%, and 99.68% under the four loads, respectively. It can achieve a high diagnostic rate under various loads, and the average diagnostic rate reaches 99.64%. When the model constructed in this paper is used for bearing fault diagnosis, its diagnostic accuracy is higher than the existing models, so it is more suitable for bearing fault diagnosis.

### 4.3. Bearing Data Validation Test at the University of Paderborn

To further verify the effectiveness of the proposed method, a validation test is carried out on the bearing data of the University of Paderborn, Germany. The test bearing model of the University of Paderborn is 6203 type rolling bearing, and the failure types are artificial damage and real damage. This verification experiment uses real damage data for testing, the sampling frequency is 64 KHZ, the rotational speed is 900 rpm and 1500 rpm, the load torque is 0.7 Nm, and the bearing radial force is 1000 N. There are only two types of faults: outer ring fault and inner ring fault, so the fault diagnosis on this dataset is equivalent to a three-category problem. As with Case Western Reserve University in the United States, 1024 consecutive points were taken as a sample data, and 750 samples were obtained at a rotational speed of 900 rpm. The same as 900 rpm, the same data set preparation method is used at 1500 rpm, and the same amount of data is obtained. The training set, test set, and fault label of the validation experiment are shown in Table 6.

**Figure 16:**
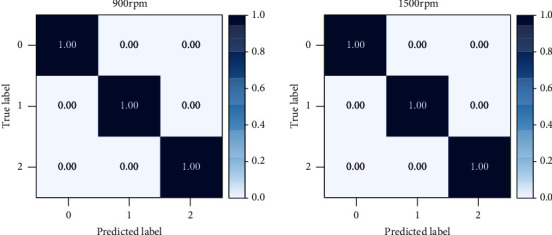
Diagnostic results of this model in bearing data at the University of Paderborn.

The structure and parameters of the model constructed in this paper are the same as those in the previous experiments, and only the number of classifications is changed from ten to three. The diagnostic results of the model on the bearing dataset of the University of Paderborn are shown in Figure 16. From the diagnosis results, it can be concluded that the diagnosis rate of this model for various bearing faults at two speeds has reached 100%. It shows that the excellent performance of this model in bearing fault diagnosis is not limited by the dataset, and it can also achieve good diagnostic results in datasets other than Case Western Reserve University.

Finally, this paper also conducts a control experiment on the bearing data set of the University of Paderborn. The average value of ten repeated experiments is used as the experimental result. The diagnostic results of each model are shown in Table 7. The average diagnostic rate of SSA-SVM is the lowest at 95.67%. The average accuracy of PSO-RF, AVSSA-KELM, and attention convolutional networks has a certain improvement compared with SSA-SVM, which can reach about 98%. The diagnostic accuracy of attention-weighted LSTM, double attention lacks EMD, and 1D-CNN-LSTM has been further improved, reaching about 99%. The diagnostic rate of the model in this paper is 100%, which is 4.33%, 2.67%, 2%, 1.99%, 1.16%, 0.73%, and 0.67% higher than the comparison models, respectively.

This model combines the advantages of CNN and LSTM on the premise that EMD reduces the noise of bearing signals and uses the attention mechanism to enhance the attention of the model to important features, which can improve the accuracy of fault diagnosis of the model and meet the accuracy requirements of bearing fault diagnosis.

## 5. Conclusion

In order to overcome the difficulty of extracting complex environmental features in traditional bearing fault diagnosis, this paper proposes a bearing fault diagnosis network with a double attention mechanism. The complex fault signal is decomposed by EMD, and the signal with a strong correlation with the original signal is screened out by the Pearson correlation coefficient method for reconstruction. Secondly, the new network is constructed by combining attention convolutional networks with LSTM. The constructed network can simultaneously extract spatial and temporal features in fault data. The traditional convolutional neural network is improved by using the spatial attention mechanism, so that it can distinguish the importance of features while retaining all feature information. Similarly, the time series attention mechanism is used to select the temporal features extracted by LSTM, which further improves the diagnostic accuracy of the model. The test of this model on the bearing data of Case Western Reserve University in the United States and the bearing data of the University of Paderborn in Germany shows that the model can accurately extract fault information, and the diagnosis rate of each load under noise interference is more than 99%. The diagnostic accuracy of the model is improved to a certain extent compared with the previous models and has good generalization ability and robustness.

The fault diagnosis model proposed in this paper has high accuracy for the detection of bearing failure, but it has not considered the operation of the bearing before fault. Therefore, the state before the bearing fault and the remaining useful life should be considered in future research to minimize the impact of bearing fault on life and production. Finally, the scope of application of the model in this paper is not only limited to the fault diagnosis of rolling bearings but also has broad application prospects for the fault diagnosis of other rotating machinery.

## Figures and Tables

**Table 1:** Experimental samples.

Fault type	Fault diameter	Training samples	Test sample	Fault label
Normal	0	364	89	0
Inner ring failure	0.1778	361	113	1
	0.3556	301	71	2
	0.5334	371	102	3

Rolling element failure	0.1778	386	89	4
	0.3556	370	104	5
	0.5334	382	93	6

Outer ring failure	0.1778	377	98	7
	0.3556	395	78	8
	0.5334	389	88	9

**Table 2:** Structural parameters of this model.

Layers	Network name	Key parameter	Output size
0	Input layer	—	1024 × 1
1	Convolutional block1	Number of neurons: 512, convolution kernel 3 × 1	512 × 1
2	Convolutional block12	Number of neurons: 128, convolution kernel 3 × 1	128 × 1
3	Spatial feature attention layer	—	128 × 1
4	LSTM1	Number of neurons: 32, dropout: 0.3	32 × 1
5	LSTM2	Number of neurons: 32, dropout: 0.3	32 × 1
6	LSTM3	Number of neurons: 32, dropout: 0.3	32 × 1
7	LSTM4	Number of neurons: 32, dropout: 0.3	32 × 1
8	Temporal feature attention layer	—	32 × 1
9	Softmax layer	—	10 × 1

**Table 3:** Comparison of ablation experiment results.

Method	Training set accuracy (%)	Test set accuracy (%)
Attention convolutional network	100	60.34
Attention LSTM	100	87.42
1D-CNN-LSTM	100	98.57
Double attention lacks EMD	100	99.24
Paper method	100	99.78

**Table 4:** Comparison of diagnostic accuracy for each model.

Method	Training set accuracy (%)	Test set accuracy (%)
SSA-SVM	90.36	70.58
PSO-RF	97.65	79.63
AVSSA-KELM	99.23	88.01
Paper method	100	99.78

**Table 5:** Comparison of accuracy rates of different models under different loads.

Method	0 hp (%)	1 hp (%)	2 hp (%)	3 hp (%)	Average accuracy (%)
Attention convolutional	74.27	60.34	87.27	77.71	74.9
Attention LSTM	82.5	87.42	84.28	84.72	84.73
1D-CNN-LSTM	96.08	98.57	98.73	97.68	97.69
Double attention lacks EMD	99	99.24	98.94	99.48	99.17
SSA-SVM	69.97	70.58	91.16	79.68	77.85
PSO-RF	80.36	79.63	93.16	93.16	86.58
AVSSA-KELM	89.18	88.01	82.49	91.46	87.79
Paper method	99.64	99.78	99.46	99.68	99.64

**Table 6:** University of Paderborn bearing experiment dataset.

Fault type	Training samples	Test sample	Fault label
Normal	200	50	0
Inner ring failure	200	50	1
Outer ring failure	200	50	2

**Table 7:** Diagnostic results of each model.

Method	900 rpm (%)	1500 rpm (%)	Average accuracy (%)
SSA-SVM	94.66	96.68	95.67
PSO-RF	98.66	96	97.33
AVSSA-KELM	98	98	98
Attention convolutional	98.67	97.35	98.01
Attention LSTM	99	98.67	98.84
Double attention lacks EMD	99.22	99.33	99.27
1D-CNN-LSTM	99.33	99.33	99.33
Paper method	100	100	100

## Data Availability

The experimental data used to support the findings of this study are available from the corresponding author upon request.
